# AAV- based vector improvements unrelated to capsid protein modification

**DOI:** 10.3389/fmed.2023.1106085

**Published:** 2023-02-03

**Authors:** Ekaterina M. Shitik, Igor K. Shalik, Dmitry V. Yudkin

**Affiliations:** Federal Scientific and Technical Program on the Development of Genetic Technologies, State Research Center of Virology and Biotechnology “Vector”, Rospotrebnadzor, World-Class Genomic Research Center for Biological Safety and Technological Independence, Kol’tsovo, Russia

**Keywords:** adeno-associated virus, inverted terminal repeats, a transgene cassette, Rep proteins, gene therapy

## Abstract

Recombinant adeno-associated virus (rAAV) is the leading platform for delivering genetic constructs *in vivo*. To date, three AAV-based gene therapeutic agents have been approved by the FDA and are used in clinical practice. Despite the distinct advantages of gene therapy development, it is clear that AAV vectors need to be improved. Enhancements in viral vectors are mainly associated with capsid protein modifications. However, there are other structures that significantly affect the AAV life cycle and transduction. The Rep proteins, in combination with inverted terminal repeats (ITRs), determine viral genome replication, encapsidation, etc. Moreover, transgene cassette expression in recombinant variants is directly related to AAV production and transduction efficiency. This review discusses the ways to improve AAV vectors by modifying ITRs, a transgene cassette, and the Rep proteins.

## 1. Introduction

Adeno-associated virus (AAV) is a non-enveloped virus, 20–25 nm in diameter, belonging to the *Parvoviridae* family. It is recognized as the leading platform for delivering genetic constructs *in vivo*. AAV is not a pathogen for human cells and cannot maintain its life cycle without helper viruses, i.e., adenoviruses (Ad) or herpesviruses. At the same time, AAV entry is independent of the cell division cycle, and the recombinant virus maintains the expression of the transgene for a long time in the form of an episome (1). Currently, there are 263 clinical trials with AAV-based therapeutics, with 32 having reached phase III (2). In addition, three FDA-approved gene therapy bioproducts based on AAV are in clinical use, such as Luxturna (voretigene neparvovec) to treat RPE65-associated Leber congenital amaurosis, Zolgensma (onasemnogene abeparvovec) to treat spinal muscular atrophy (3) and Hemgenix (etranacogene dezaparvovec) for the treatment of hemophilia B in adult patients (4). Despite such impressive results in the development of gene therapy, it is evident that AAV vectors need to be further improved. Their application in clinical trials raises concerns about low transduction efficiency and side effects resulting from the wide virus tropism and the immune response (3). There have been determined treatment-emergent serious adverse events such as hepatotoxicity, thrombotic microangiopathy in systemic delivery and neurotoxicity in CNS delivery. Moreover, 11 patient deaths among 8 trials have been reported (5). Besides, of particular interest is large-scale vector production (3). The most common approach to improving the AAV-based vector is to modify the viral capsid. Although bypassing such AAV limitations as broad tropism and ubiquitination in the cell cytoplasm (6), this approach is not sufficient to avoid the immune response to the full extent. Mutations that reduce immunogenicity always pose a risk of disrupting the receptor-binding domain and, hence, reducing transduction efficiency (7). Most importantly, capsid modification does not improve AAV production and transgene expression in the tissues of interest. These AAV enhancements can be reached by modifying the structures that directly affect the viral life cycle.

**FIGURE 1:**
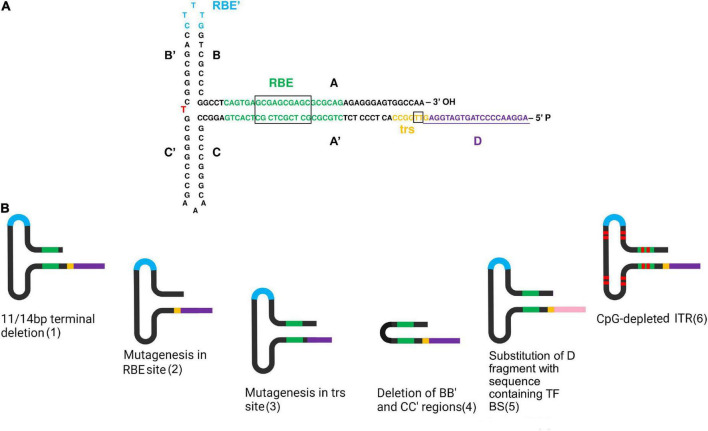
Directed structure modification of ITRs. **(A)** Detailed scheme of the ITR structure in FLIP orientation. A red letter indicates a nucleotide changing in different orientations. This scheme was created based on a figure from (110). **(B)** Some variants of ITR modification. In order from left to right: 1—11/14 bp terminal deletion does not affect AAV viability, since it is rapidly repaired during following second-strand DNA synthesis; 2—mutagenesis in the RBE site reduces binding with Rep proteins. Dinucleotide substitutions in the core 10 bp sequence decreased binding affinity over 10-fold and single-nucleotide substitution up to fivefold (43); 3—mutagenesis in the trs site decreases Rep nicking. The greatest effect of 3–10-fold Rep nicking reduction has been discovered due to mutagenesis in the core 7 bp site representing two thymines. Mutagenesis in the trs flanking regions has similarly been observed to reduce Rep nicking, but only by 20–50% (44); 4—the deletion of the BB’ and CC’ regions reduces viral productivity by 75%, meanwhile, provides an increased level of transgene expression *in vitro* and *in vivo*, reaching up to a 6.6-fold increase in some cases, compared to wild-type ITRs (47); 5—substitution of the entire D region of the 5′ ITR by a non-AAV substitute sequence containing transcription factor binding sites (TF BS) led to increased transduction efficiency, possibly due to increased transgene expression (110). Pink color indicates the sequence containing TF BS 6—CpG depleted ITRs are stable in bacterial passaging, thus facilitating AAV production (54). Red markers used for a schematic depiction of guanine and cytosine substitutions to adenine and thimine. In both pictures, the colors indicate the main structures of ITRs. Black—AA’, BB’, CC’ regions; green—RBE site with the core sequence in the black box; blue—RBE’ site; yellow—trs site with two thymines in the black box between which a single-strand break occurs; purple—D-region containing one nucleotide from trs site underlined on **(A)**. Created with BioRender.com.

The wild-type AAV genome is a linear single-stranded DNA, either positive- or negative-sensed, consisting of two genes named Rep and Cap, flanked with T-shaped inverted terminal repeats (ITRs) (8). The AAV life cycle depends on the expression of helper virus proteins, with adenovirus being the most common. Some of the Ad proteins involved, such as E1A and E2A, provide the transcription of non-structural proteins Rep78/68 from the p5 promoter and Rep52/40 from the p19 promoter (9). The Rep proteins and ITR sequences are the most significant elements in the stages of the AAV life cycle, such as replication, encapsidation, and integration into a host genome. The genome replication is initiated by Rep78/68 interaction with Rep binding sites (RBE) and terminal resolution sites (trs) in ITRs (Figure 1A). Interaction with RBE stimulates the synthesis of the DNA second strand from the free 3′-OH end of the ITRs, while nicking in the trs sequence ensures, in turn, ITR replication (10). In encapsidation, Rep52/40 binding to ITRs inserts the AAV genome into the capsid due to its helicase activity (11). The integration into the host genome also occurs due to the binding of the Rep proteins to the similar RBE sites located in the AAVS1 gene sequence as in the viral ITRs (12). In addition to the functions mentioned above, the Rep proteins regulate further AAV transcription. By repressing p5 promoter activity, the RBE-Rep complex transactivates the expression from p19 and p40 promoters (13). With the p40 promoter initiating the expression of capsid proteins named VP1, VP2, and VP3, Reps also play a crucial role in the AAV assembly (10). In contrast to wild-type viruses, recombinant AAV comprises a transgene of interest between the two ITRs. In addition to ITRs and Rep proteins, transgene toxicity and expression level also determine the recombinant adeno-associated virus (rAAV) life cycle.

To date, the research focus has been mainly placed on capsid modification. AAV capsid modifications have been widely described in previous reviews (6, 14, 15). However, there are other structures that directly affect the production of the virus, its transduction, and transgene expression. Given the strong correlation between gene therapy delivery efficiency and the viral life cycle, this review discusses the ways to improve rAAV-based vector application and production by modifying ITRs, the transgene cassette, and Rep proteins.

## 2. AAV ITR modifications

Inverted terminal repeats are complementary sequences of 145 bp that form T-shaped hairpin structures on both ends of the single-stranded AAV genome. ITRs consist of palindromic sequences of 125 nucleotides, including AA′, BB′, and CC′ regions, followed by D or D′ regions of 20 nucleotides. Within the terminal 125 bp sequence, BB′ and CC′ form two internal palindromes separated by adenine or thymine nucleotides that differ in ITR orientations (Figure 1A). The FLIP orientation has BB′ region closer to the 3′ end, and the FLOP has CC′ region at the same position (16). Two ITRs might flank the viral genome in all possible combinations: in mirrored FLIP/FLOP, FLOP/FLIP, and unidirectional FLIP/FLIP or FLOP/FLOP (17).

ITR is the only element of the AAV genome that is required in cis to produce viral particles. It is involved in numerous stages of the viral life cycle, such as replication, encapsidation, rescue (11), episomal viral genome concatemerization (18), and integration into the host genome. Each stage of the viral life cycle is significantly determined by structural ITR regions. Viral replication is provided with the free 3′-OH ends of ITRs, Rep binding sites RBE and RBE′, and trs. Being bound to the sites, as the name implies, the two largest isoforms, Rep78 and Rep 68, exhibit strand-specific endonuclease, helicase, and nickase activities (19, 20). In terms of genome encapsidation, the participation of the ITR sites in combination with Rep proteins, including Rep 52/40, is also fundamental. Some studies have considered the D region to be the most significant (11, 21, 22). Although a recent study has called into question the involvement of the D sequence in this process (23), it has been revealed that 10 proximal nucleotides of this fragment are essential for AAV rescue, replication and genome packaging. This could be attributed to partial base-pairing between the A region and the first half of the D region that stabilizes secondary structure required for trs-exposure and Rep-mediated resolution (24). Genome concatemerization is determined by the whole sequence of ITR involved in intermolecular recombination between independent viral genome (25). Interestingly, D-doubled ITR has been observed to form episomally stable concatemers *in vitro* (18, 26). Nevertheless, this effect has not been confirmed *in vivo* (27).

The significance of ITRs in the integration of viral DNA into the host genome remains ambiguous. The Rep proteins were thought to have a more significant influence on this process. At the end of the twentieth century, site-specific integration of the wild-type AAV genome was shown to occur due to the binding of Rep proteins to similar RBE sites in the cellular AAVS1 gene sequence (12). Later, a genome-wide analysis of AAV2 integration revealed that RBE sites for Rep proteins are located at numerous loci in the human genome, with the share of integration into the AAVS1 gene accounting only for 10% (28). In addition, integration into the cellular genome was observed not only for a wild-type virus but for recombinant AAV, which does not contain ORFs of Rep genes in the virions (29). However, the study on the delivery of naked linear DNA into mouse hepatocytes *in vivo* using a hydrodynamics-based transfection technique has not revealed the influence of ITRs. An insertion of linear DNA with or without ITRs occurred at a ratio of 0.3–0.5 fragments per one diploid genomic equivalent with no significant differences (27). Moreover, ITRs were observed to decrease the share of insertion in contrast with Rep proteins. During the transformation of the yeast *Saccharomyces cerevisiae*, followed by the selection on SC-lysine-uracil, genetic constructs flanked with ITRs were integrated into the genome threefold less frequently. Conversely, the expression of Rep proteins increased the AAV integration of a non-homologous fragment carrying ITRs (30). The latest studies have revealed the ITRs to be insufficient compared with the Rep proteins in binding to cellular sites of DNA damage that might be significant in AAV2 genome integration (31). Meanwhile, chromosomal rAAV integration events have been found in human hepatocytes of a xenogeneic liver regenerated in a mouse model at a frequency of 1–3% regardless of transduction methods (32).

Moreover, during cell transduction with AAV1 vectors encoding CRISPR-Cas nucleases followed by next-generation sequencing, ITRs have been observed to integrate into Cas9-induced double-strand breaks in the cellular genome in a high percentage. Significantly, the highest number of breakpoints was detected in BB′ and CC′ regions of ITRs (33).

In addition to functions directly related to the AAV life cycle, ITR sequences have been discovered to be transcriptionally active. The site of the mRNA transcription initiation has been determined to be between 109 and 145 bp, corresponding to the A and D regions of ITRs (34). Recently, the presence of mRNA transcripts for both ITRs has been demonstrated, but for the 3′ ITR significantly greater. Furthermore, the 3′ITR transcriptional activity has been observed to manifest diversely in preclinical and clinical models. In non-human primates, it caused a toxic neurological response, whereas no significant effect was observed in mice (35). Although promoter activity is a common property for the terminal repeats of different viruses (36–38), given the rapid introduction into clinical practice, the ITR activity negatively influences AAV-based gene therapy vectors (35).

Moreover, ITRs have been found to reduce AAV transduction efficiency through 16 CG-rich motifs in each repeat. CpG motifs in ITRs, as well as in a transgene cassette, initiate TLR9-dependent innate and adaptive immune responses resulting in detectable loss of transgene expression (39). It is worth noting that the motifs influence not only full-genome AAV particles but also empty capsids. Empty AAV capsids have been revealed to be packaged with short DNA fragments containing numerous ITR sequences (40). Some truncated genomes containing ITRs combined with part of the AAV genome have been observed to positively affect AAV production (23).

In view of the strong interaction between the structure and functions of ITR sequences, its modification is a reasonably complicated task (Supplementary Table 1). To date, two directions of ITR modifications can be distinguished: directed structure modification (Figure 1B), the most prevalent direction, and the combination of the ITRs obtained from different AAV serotypes.

Directed structure modification is restricted by ITR variability since it is a palindromic GC-rich sequence. Development of sequencing methods of ITRs has revealed single nucleotide substitution and deletion in its sequences occurring apparently during the process of plasmid production (17). Interestingly, it has been demonstrated that an 11 bp terminal deletion in 3′ITR (41) and a 14 bp terminal deletion at each ITR (42) do not affect viability and could be used in AAV production. Furthermore, ITRs carrying large mutations have been observed to regain their sequences except for the cases when one AA, BB′, CC′ region forming a hairpin with one D-sequence was present, only one AA′, BB′, CC′ region at all was present, or only two D-sequences were present. It is important to note that in the absence of rescue of the AAV genome, the last two cases allowed replication of the original plasmid (21). Despite the ability of ITRs to self-correct, directed modifications can negatively influence AAV production. For instance, substitutions in the RBE site located in the A region of ITRs have been shown to reduce binding with Rep proteins. For the entire 22 bp sequence, the most significant effect has been revealed for the core 10 bp sequence, in which dinucleotide substitutions decreased binding affinity over 10-fold and single-nucleotide substitution up to fivefold (43). Concurrently, Rep nicking also might be affected by directed mutagenesis in the trs sites. The greatest effect of 3–10-fold Rep nicking reduction has been discovered due to mutagenesis in the core 7 bp site representing two thymines between which a single strand break occurs. Mutagenesis in the trs flanking regions has similarly been observed to reduce Rep nicking, but only by 20–50% (44). In addition, the introduction of longer fragments of 8 bp of endonuclease restriction site *Hpa*I into the flanking region of the trs site has been discovered to decrease Rep nicking 100-fold (45) without affecting targeted integration (46).

The deletion of the BB′ and CC′ regions also has a significant impact on AAV production. Modified U-shaped ITRs with A and A′ regions connected with five nucleotides have been found to reduce viral productivity by 75% without affecting genome encapsidation. At the same time, the U-shaped ITRs have provided an increased level of transgene expression *in vitro* and *in vivo*, reaching up to a 6.6-fold increase in some cases, compared to wild-type ITRs (47).

Despite such limitations affecting the AAV life cycle, some modifications have been demonstrated to improve the virus transduction. A modified ITR of 165 bp flanked with a double-stranded D regions consisting of D and complementary D′ sequences in the original plasmid have been discovered to be sufficient for AAV replication (48). Meanwhile, such modification in one ITR combined with a complete deletion of the D-sequence in another ITR resulted in single-polarity AAV vectors being formed (49). Substitution of the entire D region of the 5′ ITR by a non-AAV substitute sequence containing transcription factor binding sites led to increased transduction efficiency, possibly due to increased transgene expression (50). Interestingly, during the AAV production in the baculovirus/Sf9 cell system, the insertion of additional D-sequence back-to-back has caused a decrease in the viral titer and a 10-fold reduction in rAAV genome encapsidation. At the same time, the addition of baculovirus enhancer elements to the modified ITRs restored the level of full-genome viral particles (42).

Directed structure modification of ITRs can also improve the properties of self-complementary AAV (scAAV). Unlike wild-type viruses, scAAV has a double-stranded genome that considerably increases the transduction efficiency *in vitro* and *in vivo*. The scAAV genome includes the two ITRs flanking a transgene cassette and the third middle ITR dividing two strands of a transgene. Deleting the trs site alone or in combination with the D-sequence in the middle ITR has led to avoiding Rep-nicking and increasing the level of full-genome encapsidation in scAAV production (51, 52). It should be emphasized that substituting the full middle ITR with a short hairpin DNA led to functional viral particles being formed, with their efficiency comparable to scAAV (53).

A modification of the ITR structure was also performed to avoid an immune response. 15% nucleotides in the ITRs of AAV serotype 2 were modified, reducing GC content to 60%. The selection of substitutions was carried out on the alignment of ITR sequences of different serotypes, including AAV1, AAV2, AAV3, AAV4, AAV6, and AAV7. As a result, the AAV particles containing CpG-free ITR were revealed to have a threefold reduction in capsid yield and the same percentage of empty capsids compared to wild-type viral vectors. Transduction of the AAV vectors carrying a micro-dystrophin expression cassette *in vivo* demonstrated no difference between CpG-free and wild-type ITRs (54). Therefore, although AAV vectors with CpG-depleted transgene cassettes were observed to improve the efficiency of transduction in different ways (39, 55), CpG motif depletion in ITRs had no such effect. Nevertheless, the CpG-free ITRs were demonstrated to be stable in bacterial passaging, thus facilitating AAV production (54).

The second line of ITR modification, based on different serotype comparisons, has been used not only to develop CpG-free repeat sequences. Although the ITR of AAV2 (ITR2) is the flanked region most commonly used for different serotypes due to its compatibility (56), this approach is not always feasible. For instance, a combination of Rep3 with ITR3 in AAV serotype 3 production was observed to increase viral titer and improve transduction efficiency in human hepatocellular carcinoma cell lines *in vitro* fourfold compared to Rep2 and ITR2 (57). Therefore, the AAV clinical application based on ITR2 might be insufficient and requires developing new combinations of ITRs.

To date, the available combinations of ITRs have been derived from AAV2, the most studied serotype, and AAV5, the most distinct from all other serotypes. Previously, ITR2 and ITR5, although having conserved sequences, were demonstrated to bind to the Rep proteins corresponding to the serotype (58). Despite that fact, Yan et al. have developed hybrid rAAV vectors carrying ITR2 and ITR5 simultaneously (AAV2:5 ITR) that are effectively packaged into AAV2 and AAV5 capsids. Compared to AAVs with original ITRs, AAV2:5 ITR was found to be more efficient at a lacZ gene reconstitution from minigenes *in vitro* in HeLa cell line, primary fetal fibroblasts (59), and *in vivo* in mouse skeletal muscle, liver, and heart (60). In addition, a chimeric ITR consisting of the ITR5 15 bp sequence from the AA′ region and ITR2 nicking stem was obtained. The chimeric ITR origin was revealed to be functional for replication with both Rep2 and Rep5 (61). Interestingly, ITR5 has been shown to have a significant capacity to produce various AAV serotypes. The AAV1–AAV6 vectors obtained with ITR5 have a higher efficiency of gene expression than the same vectors obtained with ITR2 (62).

The ITR study is not limited to serotypes 2 and 5. ITRs of different serotypes were demonstrated to have different transcriptional activities, with the transcription start sites differently located in the RBE site. The highest transgene expression was discovered for ITR2 and ITR3, the intermediate for ITR4, and the lowest for ITR1 and ITR6. In contrast to the previous cases with a constant expression level in different cells, ITR7 was observed to have cell-specific promoter activity (63). Given that the similar transduction efficiency of recombinant AAVs with ITRs of different serotypes *in vivo* was previously shown (64), the further study can serve as a basis for developing rAAV vectors comprising new hybrid or new combination of ITRs with which to avoid side effects caused with the ITR promoter activity.

## 3. Transgene cassette modifications

In contrast to wild-type viruses, rAAV vectors comprise a transgene of interest flanked with a non-native promoter and poly(A) signal. The p5 promoter is transferred to a separate plasmid for Rep transcription, while the relatively weak AAV poly(A) signal is substituted with a stronger one (65). Depending on the degree of cytotoxicity and immunoreactivity, expression of the transgene of interest can impair the assembly of viral particles in packaging cells and reduce the transduction efficiency both *in vitro* and *in vivo*. Modifying the flanking regulatory sequences holds great potential to solve the problem through selective transgene expression in specific cells and tissues or temporary transgene silencing.

One modification is selecting an appropriate promoter to directly determine the transcription level of the transgene of interest. Promoter selection is significantly limited by the sequence length because AAV vector genome capacity should not exceed 4.7 kb (66). Previously, transcription control of GDNF ORF delivered with an AAV1 vector was performed with a bidirectional tetracycline-responsive promoter in combination with a reverse tetracycline transactivator. As a result, doxycycline-treated rats were shown to reach biologically active concentrations of GDNF in the striatum in contrast to uninduced controls (67). In addition, an enhancer from jaagsiekte sheep retrovirus consisting of the shortest JE sequence and the U3 region of the long terminal repeat has been applied in AAV vectors in combination with the chicken beta-actin promoter. Although having a lower level of transgene expression than the CAG promoter, the hybrid promoter demonstrated non-cardiac and respiratory tract- and liver-specific activity (68).

Another option to control transgene expression is to use the Cre/loxP system that is applied to insert, delete or invert DNA sequences between loxP sites. Despite widespread use, the system in AAV vectors has revealed off-target Cre-independent transgene expression in wild-type C57BL/6J mice (69). For improving Cre-dependent vector transduction to neurons, tTARGIT AAVs containing Flp-dependent tetracycline transactivator and tetracycline response-driven Cre-dependent elements were developed to selectively express transgene of interest in particular neuron cells *in vivo* (70).

Post- transcriptional regulation by microRNAs (miRNAs) is also used to control transgene expression (Supplementary Table 2). miRNAs are a class of small non-coding RNAs of approximately 22 bp length that negatively regulate gene expression by directing Argonaute proteins to target sites in the 3′ untranslated region of mRNAs. Given the tissue-specific expression of miRNAs (71), such an approach is a great selective option for rAAV vectors. It is worth noting that wild-type AAV expresses its own small non-coding RNAs. Hairpin-formed sequences, such as the trs site in the ITR, p5, and p40 promoters, are a source of small non-coding RNAs that could regulate p5 promoter activity and have the potential to inhibit adenoviral infection. Despite influencing viral gene expression, AAV small RNAs have not been shown to affect cellular miRNA expression (72).

The most studied microRNA is miR122, with its target sites integrated into the 3′UTR of rAAV vectors transgene cassette. miR-122 has been studied on rAAV hepatic de-targeting since it represents the largest share among all liver miRNAs, accounting for approximately 70%. Integration of its five target sites into the 3′UTR of rAAV vectors has been observed to reduce liver expression of luciferase by 50-fold and LacZ expression by 70-fold in a mouse model. Interestingly, this insertion was shown to increase luciferase activities and LacZ expression in mouse cardiac muscle with no effect on skeletal muscle (73). Further research on the construct delivery to the heart muscle also yielded positive results *in vitro* and *in vivo.* Cardio-specific expression with no hepatic side effects was achieved with the insertion of miR-122 target sites in combination with the most cardiotropic serotype AAV9 and hybrid promoter containing the cytomegalovirus enhancer with a cardiac myosin light chain promoter (74). Similar research was conducted to increase the specificity of the leptin sequence delivery in the AAV2/8 vector to adipose tissue. Selective adipose gene transfer was reached using the adiponectin promoter activity in combination with miR-122-mediated post-transcriptional regulation (75). This approach has also been shown to be effective in tumor-selective transduction. Insertion of miR-122 target sites in the 3′UTR of the AAV8 vectors containing herpes simplex virus-thymidine kinase suicide gene led to 7-reduction in the hepatocellular carcinoma growth without liver toxicity in a murine model (76).

Not only hepatic de-targeting but also transgene silencing in skeletal muscle were studied to increase cardiac-specific expression of AAV9. To achieve this goal, the binding site of miR-206 was inserted into the 3′UTR of the rAAV vector genome. During the study, the suppression of transgene expression was observed in mouse skeletal and cardiac muscles due to the cross-reactivity of these sites with miR-1. The problem was solved by substituting the cytosine located in the third position in the 3′ end with guanine in a seed-matched sequence of the target site. Moreover, eight other single-nucleotide substitutions were identified with the potential to improve the inhibitory activity of miR-206 (77). The modified miR-206 binding sites were used in further studies. A combination of miR-122- and miR-206- mediated post-transcriptional regulation was used in AAV8 vector delivery in rhesus macaques. After intramuscular administration, hepatic and skeletal muscle distribution was observed to be higher in the macaque than in mice, possibly due to higher vector doses (78). Furthermore, despite earlier results, the most recent studies have demonstrated the AAV9 regulated by miR-122- and miR-206 vectors to be an ineffective heart-specific delivery system. This result was attributed to the significantly impaired miR-122 expression detected in the murine heart tissue. Since such a result was also detected in the heart tissues of patients with cardiomyopathy and human induced pluripotent stem cell-derived cardiomyocytes (79), this approach should be further improved.

It is worth noting that the neurotropic AAV9 vectors regulated with miR-122 and miR-1 have recently been discovered to have five times greater suppression of expression in the heart compared to the liver (80). Nonetheless, the integration of binding sites of miR-1d alone was sufficient to avoid cardiotoxicity (81). In addition to the miRNA mentioned above, miR-208a post-transcriptional regulation accompanied by specific promoter activity has also been noticed to prevent heart side effects (82).

The miRNA regulation was performed to avoid immune response to rAAV vectors. Despite the negative results obtained earlier due to promoter shut-off (73), the expression of highly immunogenic transgenes was effectively inhibited in antigen-presenting cells. The human alpha-sarcoglycan gene silencing was achieved with the rAAV1 vectors, including miR142-3p target sites in control C57BL/6 mice. However, these results have not been reproduced in sarcoglycan-deficient dystrophic mice through CD4 and CD8 T-cell responses (83). The approach was applied in C57BL/6 mice for ovalbumin delivery in skeletal muscle. Insertion of miR-142-3p target sequences provided a prolonged transgene expression without cellular infiltrate in the injection area (84). In addition, the incorporation of miR-142-3p binding sites was also detected to be effective in reducing the ovalbumin-specific IgGs response, with the differences between murine strains in immunosuppression of ovalbumin also identified (85). The modified rAAV1 vectors were also improved with the insertion of miR-652-5p binding sites. miR-142/652-5p regulated vectors repressed ovalbumin expression in dendritic cells and macrophages with no effect on myoblasts. Novel vectors were revealed to be more effective in reducing the cytotoxic CD8 + T cell and macrophage infiltration than the previous ones (86).

miRNA-regulated AAV vectors have been applied to other target tissues. Integration of miR-181 binding sites in combination with human rhodopsin kinase promoter activity was found to improve the specificity of AAV2 vector delivery in the retina. In intravitreal injection, the modified vectors were detected to suppress transgene expression in all cells except photoreceptors (87). Additionally, the miR-183-regulated AAV9 vector was found to improve the central nervous system targeting in non-human primates. Compared to the unmodified vectors, the vectors containing miR-183 binding sites in the 3′UTR reduced dorsal root ganglion toxicity at a statistically significant level (88).

In addition to reducing the off-target transgene expression, miRNA regulation was found to increase AAV2/2, AAV2/5, and AAV2/8 vector production. Initially, a decrease in transgene expression in HEK293 was achieved by introducing the target sites of miR-20a, miR-21, and miR-222, which are naturally expressed in this cell line. More significant suppression of transgene expression in HEK293 was achieved by integrating target sites of miR-122a, miR-367, and miR-373 into the 3′UTR of the AAV genome in combination with transiently over-expressed corresponding miRNAs. Reduced transgene expression proved to be directly correlated with increased efficiency of AAV particle assembly, reaching a maximum for highly toxic transgenes (89).

Interestingly, miRNA has also been applied to provide accurate processing of short hairpin RNAs (shRNAs) in AAV vectors. miR-33 as a pre-miRNA scaffold for shRNAs has been demonstrated to prevent the formation of truncated AAV genomes and achieve effective gene silencing in HEK293 cells and mice (90). Furthermore, shRNAs alone have been observed to improve AAV production. Cotransfection of shRNA coding cassettes with the AAV plasmid system has caused the increased AAV protein expression detected with Western blot analysis, followed by a 10-fold increase in viral vector yields (91).

Besides the approaches mentioned above, there are some other promising modifications of a transgene cassette, such as riboswitch-regulated AAV vectors. Integration of off-switched guanine-responsive aptazyme into the AAV genome containing highly toxic transgene was discovered to improve vector yield up to 23-fold. Compared to unmodified constructs, the riboswitch-regulated vectors demonstrated lower transgene expression after transduction which was compensated by applying a 1.5-fold higher dose. It is worth noting that only 3′ integrated aptazymes provided dose-dependent regulation of transgene expression in the packaging cells (92). These results were reproduced in the transduction of murine models *in vivo* (93). The dose-dependent regulation was also achieved by developing a new riboswitch representing a morpholino-responsive hammerhead ribozyme. Besides a high ability to be modulated, this on-switched system can improve the AAV-delivered transgene expression up to 200-fold (94).

In addition to miRNA binding sites and riboswitches, translation inhibition was examined to increase AAV vector titers through transgene silencing. A tryptophan RNA-binding attenuation protein (TRAP) was applied in combination with the TRAP-binding sequence inserted upstream of the ribosome entry site in the AAV genome. Despite 100-fold suppression of the transgene expression in contrast to unmodified vectors, no improvement in AAV titers was detected (95). Transgene separation in unfunctional sequences was applied to avoid transgene toxicity during AAV production. An efficient transgene sequence restoration *in vivo* was revealed for trans-splicing (96) and intron-splicing mechanisms. Meanwhile, the last-mentioned approach was used with baculovirus expression vector systems to produce AAVs (97).

To conclude, modification of the transgenic cassette is a tremendous area for significant improvements not only in AAV production but also in the efficiency of target organ transduction.

## 4. Rep protein modifications

A critical step in the AAV life cycle is genome replication. This stage is determined by two AAV main structures, the Rep proteins and the ITRs located at the ends of the viral genome. The Rep open reading frame (ORF) of AAV encodes four proteins named according to their molecular weights of 78, 68, 52, and 40 kDa. Each pair of Rep78/68 and Rep 52/40 includes a full-length variant, with a truncated one formed from a bifunctional transcript with intron excising in splicing, respectively. The Rep78/68 expressed from the p5 promoter play a significant role in viral DNA replication (98), while the Rep52/40 transcribed from the p19 promoter function mainly as DNA helicases and contribute to the accumulation of single-stranded progeny of the virus (99). Genome replication is defined by large Rep protein binding to duplex ITRs in a site- and strand-specific manner (100). By binding with the RBE sites representing a tandem repeat of the GAGY motif, Rep78/68 triggers the duplication of the AAV genome (43, 45, 101, 102). After AAV genome copying, Rep78/68 activates ITR replication through a single-strand cleavage reaction in the trs site. Integration into a host genome is also provided with the Rep DNA binding sites located in a region on the q-arm of human chromosome 19 (98, 103, 104). Given the significant role of the Rep in the AAV life cycle, a high degree of Rep and ITR gene similarity was revealed among the different AAV serotypes. The AAV2, −3, −4, and −6 Rep proteins were found to be approximately 90% identical, except for the share containing conservative amino acid substitutions. This similarity provided the basis for developing an approach of cross-complementation between Rep and ITR genes of different serotypes, such as cross-packaging and pseudotyping. The relevance of Rep proteins stems not only from their participation in genome replication but also from their influence on viral genome regulation. In terms of gene expression regulation, Rep proteins act as activators and repressors. In addition to regulating transcription from the p5, the p19, and the p40 promoters, the Rep inhibits the heterologous promoter activity of helper viruses and proteins involved in the G1 phase of the cell cycle (105). Considering the participation of Rep in AAV genome replication, encapsidation, and regulation of gene transcription, their modifications are of particular interest (Supplementary Table 1).

One Rep modification was discovered during AAV cross-packaging studies. Rep proteins of AAV2 (Rep2) are commonly used in this process due to their ability to package viral genome into the capsid of other serotypes. Rep2 efficiency has been confirmed in the cross-packaging of AAV1, AAV6, AAV8, AAV9, and AAV10. Rep proteins of AAV8 were demonstrated to decrease VP expression significantly compared to Rep2. Given the difference in the p40 promoter, the 3′ end of Rep8 was substituted with the same sequence of Rep2. The substitution not only restored the high expression of the capsid proteins but also demonstrated a higher packaging efficiency of vector genomes. These new Rep hybrids increased the percentage of “complete” capsids by about 2–4 times for all non-AAV2 serotypes tested. This approach is expected to provide higher rAAV titers, thereby facilitating the expansion of gene therapy based on the AAV (106).

Another modification is connected with the fact that the assembly of vectors based on AAV might be limited by the ability of Rep78 to inhibit adenoviral replication. A computational reengineering of the Rep gene with the recoding of synonymous codon pairs was used to address this limitation. While preserving the amino acid sequence, the Rep genomic segment was recoded to disrupt any cis-acting sequences that might inhibit the Ad function. One of the Rep mutants was constructed by rearranging synonymous codon pairs while conserving the free energy of the folded RNA to prevent significant changes in the secondary structure. In parallel, by including underrepresented codon pairs, the Rep gene was constructed with a maximally deoptimized codon. Both resulting genes were created using *de novo* genome synthesis and retained the polypeptide chain and endonuclease properties of Rep78. At the same time, reengineering a pair of synonymous codons enabled overcoming the inhibitory effects of Rep proteins on Ad replication. The two modified mutants dramatically increased Ad replication and viral titer yield to levels corresponding to Rep-negative Ad. This observation provides insight into the molecular interactions of AAV Rep and Ad replication, extending the applicability of synonymous codon pair reengineering in AAV production (107).

The next objective of Rep modifications was to improve AAV production in insect cells in the baculovirus-mediated system. Despite the efficiency of this production method, the Bac-Rep construct exhibited genetic instability upon serial passage represented in tandem duplication of homologous regions of the Rep78 and Rep52 genes. The genetic abnormality decreased AAV vector yields in large-scale rAAV production. To solve this problem, the Rep gene was modified to encode a bifunctional mRNA transcript. Synthesis of Rep78 and Rep52 AAV polypeptides was performed with a “leak” mechanism that enables the expression of Rep and Cap proteins from a single mRNA. For optimizing the translation of Rep78, its start codon and 10 additional downstream triplets were mutated. In addition to the Rep78 start codon modification, the CCG codon encoding the proline at position 2 of the Rep78 amino acid was substituted with the GCG (alanine) codon to maintain the integrity of the Kozak sequence. Simultaneously, substitutions below the initiation codon Rep78 were made to facilitate efficient ribosomal scanning. They were made in such a way that AUGs lying outside the ORF encoding Rep52 gave a silent mutation, and those lying inside gave a conservative amino acid substitution from leucine to methionine. These modifications improved the stability of the Bac-Rep construct and increased overall system robustness (108).

To increase the efficiency of Rep, the ORF sequence was modified, and the influence of the promoter was studied. Previously, attempts were made to modify AAV by replacing the p5 promoter with the simian virus 40 (SV40) promoter to increase the number of Rep proteins, but this approach was not successful (58). Moreover, to increase Rep expression, AAV helper plasmids were constructed containing various strong heterologous promoters replacing the p5 promoter in the AAV cassette. Despite achieving a high level of Rep expression, the application of plasmids spCMV/AAV and pHIV/AAV gave the lowest yields of rAAV. In contrast, the application of the attenuated p5 promoter gave the highest rAAV yields. Apparently, suppression of the Rep 78/68 protein by an ineffective translation start signal (ACG) resulted in increased expression of the AAV capsid protein, which contributed to higher titers of the rAAV vector. Consequently, the presence of strong heterologous promoters in helper plasmids could reduce the efficiency of vector DNA replication. These results strongly suggest that highly regulated expression of the Rep protein is required to obtain optimal levels of rAAV vectors (109).

Taken together, the studies referred to above indicate that modification of Rep proteins is a prospective way to improve rAAV production.

## 5. Discussion

Modifying the structures determining the viral life cycle is seen to significantly improve AAV production, transduction efficiency, and transgene expression in tissues of interest. The study of ITRs has resulted in increased AAV production. Modified ITRs, especially those containing mutated D-regions (48, 50), were discovered to improve AAV replication compared to wild-type ITRs. By applying a similar modification, AAV production has been adapted not only in mammalian cells but also in the baculovirus/Sf9 cells system (42). Moreover, modification of ITRs has further improved the scAAV, which, due to the double-stranded genome, significantly increased the transduction efficiency *in vitro* and *in vivo* (51–53). While maintaining the transduction efficiency comparable to wild-type viruses, AAV production has been facilitated by CpG-free ITRs (54). In addition to AAV production, modification of ITR has advanced the cross-reactivity of AAV different serotypes (59–61). The selection of regulatory elements in the transgene cassette also has great potential for improving AAV-based vectors. By influencing direct transgene expression, the regulatory elements increase the viral vector yields in packaging cells and the efficiency of transduction both *in vitro* and *in vivo*. Multiple approaches are currently being used, including ones on non-human primates. Relevant results have also been achieved in Rep protein modification. Hybrid Reps have been obtained that promote AAV vector assembly by reducing the share of empty capsids (106). Through regulation of Rep transcription and translation, AAV assembly has been enhanced both in mammalian and in Sf9 insect cell lines (58, 109).

Thus, modification of ITRs, a transgene cassette, and Rep proteins are promising directions for AAV vector enhancement. The main disadvantages of the ITR and Rep protein directions are their structure-function relationship, however, unlike modifications of capsid proteins, they have a stronger impact on the AAV life cycle, making them indispensable in this field. Further fundamental research in this area will improve the production and the application of AAV-based vectors through effect on replication, rescue and encapsidation. Study of possibilities to control transgene expression is also highly relevant, however, as is evident from our review, to date there have already been a lot of approaches used in the vector development. A comprehensive approach incorporating these modifications will allow a new generation of AAV-based vectors to be created with an improved method of production and with reduced side effects.

## Author contributions

ES and IS prepared the final manuscript. ES analyzed AAV ITR modification and transgene modification. IS analyzed Rep protein modification. DY supervised the project and reviewed and edited the manuscript. All authors contributed to the article and approved the submitted version.
