# Supplementary material for: AAV- based vector improvements unrelated to capsid protein modification
Source: Front Med (Lausanne). 2023 Feb 3;10:1106085. doi: 10.3389/fmed.2023.1106085 (PMC9935841; doi:10.3389/fmed.2023.1106085)
Supplement: Supplementary file 1 [file Table_1.docx]

Supplementary Material

**Supplementary table 1: Effects of modifications of ITRs and Rep proteins on rAAV production**

| **Name of structure** | **Effect on rAAV production** | **Modification** | **Particular mechanism and additional effect** | **Reference** |
| --- | --- | --- | --- | --- |
| ITRs | Increase | Deleting the trs site alone or in combination with the D-sequence in the middle ITR of the scAAV | Avoiding Rep-nicking and increasing the level of full-genome encapsidation | 51, 52 |
|  |  | Native ITRs | A combination of Rep3 with ITR3 in AAV serotype 3 production increased viral titer and improved transduction efficiency compared to Rep2 and ITR2. | 57 |
|  | Decrease | Mutagenesis in the RBE site | Disrupts the replication and encapsidation through reducing binding with Rep proteins. | 43 |
|  |  | Mutagenesis in the trs site | Disrupts the replication through the disruption of Rep nicking | 44 |
|  |  | The introduction of fragments of 8 bp into the flanking region of the trs site | Disrupts the replication through the disruption of Rep nicking. Doesn’t affect targeted integration | 45 |
|  |  | The deletion of the BB′ and CC′ regions | Reducing viral productivity by 75% without affecting genome encapsidation. Increased level of transgene expression compared to wild-type ITRs | 47 |
|  |  | CpG-free ITRs | Despite 3-fold reduction in capsid yield, CpG-free ITRs were demonstrated to be stable in bacterial passaging | 54 |
|  |  | The insertion of additional D-sequence back-to-back | Caused a decrease in the viral titer in the baculovirus/Sf9 cell system | 42 |
|  | Neutral | One modified ITR of 165 bp flanked with a double-stranded D regions consisting of D and complementary D′ sequences in the original plasmid | Sufficient for AAV replication | 48 |
|  |  | Double-D ITR combined with a complete deletion of the D-sequence in another ITR | Single-polarity AAV vector production | 49 |
|  |  | Substitution of D sequence with sequence containing transcription factor binding sites | Increased transduction efficiency, possibly due to increased transgene expression | 50 |
|  |  | Substitution of the full middle ITR in scAAV with a short hairpin DNA | Formed functional viral particles | 53 |
|  |  | Chimeric ITR consisting of ITR5 and ITR2 | Significant for cross-packaging. Revealed to be functional for replication with both Rep2 and Rep5 | 61 |
| Rep proteins | Increase | Hybrid Rep consisting of Rep 2 and Rep8 | Restored the high expression of the capsid proteins. Increased the percentage of "complete" capsids by about 2–4 times for all non-AAV2 serotypes. | 106 |
|  |  | The Rep gene encoded a bifunctional mRNA transcript. Mutated Rep78 start codon and 10 additional downstream triplets | Solved the problem of tandem duplication of the Rep78 and Rep52 homologous regions formed upon serial passage in the baculovirus/Sf9 cell system | 108 |
|  |  | Attenuated p5 promoter with ACG translation start signal | Increased expression of the AAV capsid protein | 109 |
|  | Neutral | The Rep mutants constructed by rearranging synonymous codon pairs | Overcome the inhibitory effects of Rep proteins on Ad replication. | 107 |

**Supplementary table 2: Post-transcriptional regulation with microRNA binding sites used to control transgene expression in rAAV-based vectors**

| **Name of miRNA** | **The purpose of the modification** | **Results** |
| --- | --- | --- |
| miR-122 | Hepatic de-targeting | 1. Reduction in liver expression from 50-fold to 70- fold in a mouse model (73); 2. Cardio-specific expression with no hepatic side effects in mice. It was applied in combination hybrid promoter containing the CMV enhancer with a cardiac myosin light chain promoter (74) 3. Selective adipose gene transfer was reached in combination with the adiponectin promoter activity in adipose tissue (75) 4. Tumor selective transduction in hepatocellular carcinoma was reached without liver toxicity in a murine model (76). |
| miR-122/206 (modified) | Transgene silencing in liver and skeletal muscle | 1. After intramuscular administration, hepatic and skeletal muscle distribution was observed to be higher in rhesus macaque than in mice, possibly due to higher vector doses (78) 2. It has been shown to be an ineffective heart-specific delivery system in the murine heart tissue, the heart tissues of patients with cardiomyopathy and human induced pluripotent stem cell-derived cardiomyocytes (79) |
| miR-1d | Cardiac muscle de-targeting | Efficient to avoid cardiotoxicity (81) |
| miR-208a | Cardiac muscle de-targeting | Noticed to prevent heart side effects (82) |
| miR 142-3p | Inhibition of immune response to rAAV vectors through the control of transgene expression in antigen-presenting cells | 1. Prolonged ovalbumin transgene expression in skeletal muscle without cellular infiltrate (84) 2. Reducing the ovalbumin-specific IgGs response in mice (85) |
| miR-142/652-5p | Inhibition of immune response to rAAV vectors through the control of transgene expression in antigen-presenting cells | Repressed ovalbumin expression in dendritic cells and macrophages. Reducing the cytotoxic CD8+ T cell and macrophage infiltration more effectively that miR 142-3p binding site alone (86) |
| miR-181 | Selective expression in photoreceptors | In transgenic mice in combination with human rhodopsin kinase promoter activity improves the specificity of AAV2 vector delivery in the retina (87) |
| miR-183 | Dorsal root ganglion de-targeting | Improve the central nervous system targeting in nonhuman primates (88) |
| miR-20a, miR-21, and miR-222 | Increase vector production in HEK293 | A decrease in transgene expression in HEK293 was achieved (89) |
| miR-122a, miR-367, and miR-373 | Increase vector production in HEK293 | Binding sites of these microRNAs were integrated in the 3’UTR of rAAV transgene cassette and were used in combination with transiently over-expressed corresponding miRNAs. More significant suppression of transgene expression in HEK293 than previous one was achieved (89). |
